# Data on free amino acid contents in Japanese basket clams (*Corbicula japonica*) from Lake Abashiri and Abashirigawa River

**DOI:** 10.1016/j.dib.2017.11.075

**Published:** 2017-11-27

**Authors:** Hisaki Enda, Yoshimasa Sagane, Yozo Nakazawa, Hiroaki Sato, Masao Yamazaki

**Affiliations:** a Kohken Food and Flavor Co. Ltd., 2-3-19 Takashima, Nishi-ku, Yokohama 220-0011, Japan; b Department of Food and Cosmetic Science, Faculty of Bioindustry, Tokyo University of Agriculture, 196 Yasaka, Abashiri, Hokkaido 099-2493, Japan

**Keywords:** Free amino acid, Japanese basket clams, Habitat

## Abstract

This data article provides the free amino acid contents of Japanese basket clams from different areas of Lake Abashiri and Abashirigawa River, which belong to the same water system. Abashirigawa River flows into the south side of Lake Abashiri and then runs out from the northeast side of the lake. Clams were caught in three different areas in Lake Abashiri (Memanbetsu, Yobito, and Katayama) and from one site at Abashirigawa River (Sancho). Free amino acids in the edible portion of the clams were analyzed using an automated amino acid analyzer. The clams showed high contents in β-alanine, alanine, glutamic acid, and glycine. The clams caught in the river contains relatively higher amino acid contents. The analyzed data are provided in table format.

**Specifications table**

**Table:** 

Subject area	Agricultural science
More specific subject area	Food chemistry
Type of data	Table
How data was acquired	Free amino acid contents were measured using an automated amino acid analyzer (LaChrom Elite, Hitachi High-Technologies Corp., Japan).
Data format	Raw, analyzed
Experimental factors	Japanese basket clams were harvested at Abashirigawa River and at three different areas in Lake Abashiri.
Experimental features	Free amino acid analysis
Data source location	Lake Abashiri and Abashirigawa River (city of Abashiri and town of Ohzora, Hokkaido, Japan)
Data accessibility	Data are presented in this article.

**Value of the data**

• The data presented will be useful for nutrient assessments of Japanese basket clams.
• The data will be available for use when comparing the free amino acid compositions of clams caught from other areas.
• The data presented will be available to consider the effect of environmental factors and habitat on amino acid metabolism in these clams.

## 1 Data

Free amino acid contents of Japanese basket clams were measured using an automated amino acid analyzer. The contents of each type of free amino acid are represented in units of mg/10 g of clam wet weight, and the percentage of each amino acid relative to total amino acids was determined. Table 1 provides the free amino acid contents in the refrigerated clams, whereas Table 2 provides those measured in fresh materials.

**Table 1:** Free amino acid contents in Japanese basket clams harvested at Yobito, Lake Abashiri and Sancho, Abashirigawa River.

Amino acid	Yobito (Lake Abashiri)		Sancho (Abashirigawa River)	
	mean ± standard deviation (mg/10 g edible portion)	% of total amino acids	mean ± standard deviation (mg/10 g edible portion)	% of total amino acids
Taurine	0.43 ± 0.08	1.05	1.21 ± 0.30	0.90
Aspartic acid	0.72 ± 0.14	1.75	2.77 ± 0.70	2.06
Threonine	2.53 ± 0.27	6.17	7.18 ± 1.74	5.35
Serine	0.42 ± 0.07	1.01	2.65 ± 1.31	1.97
Asparagine	n.d.	n.d.	n.d.	n.d.
Glutamic acid	4.75 ± 0.58	11.57	16.48 ± 3.35	12.28
Glutamine	0.26 ± 0.07	0.64	3.13 ±± 0.81	2.33
Proline	n.d.	n.d.	2.17 ± 3.75	1.61
Glycine	4.19 ±	10.19	10.50 ± 2.48	7.82
Alanine	8.57 ± 1.17	20.85	55.06 ± 6.28	41.00
Citrulline	n.d.	n.d.	n.d.	n.d.
Valine	2.69 ± 0.30	6.54	7.80 ± 1.70	5.81
Cystine	0.42 ± 0.14	1.03	0.39 ± 0.07	0.29
Methionine	0.14 ± 0.04	0.35	0.13 ± 0.12	0.10
Isoleucine	1.48 ± 0.18	3.61	4.58 ± 0.89	3.41
Leucine	0.26 ± 0.05	0.63	1.04 ± 0.09	0.78
Tyrosine	n.d.	n.d.	n.d.	n.d.
β-alanine	11.71 ± 0.80	28.50	16.63 ± 5.03	12.39
Phenylalanine	n.d.	n.d.	n.d.	n.d.
GABA	2.26 ± 0.16	5.51	1.88 ± 0.12	1.40
Ornithine	0.24 ± 0.24	0.59	0.60 ± 0.34	0.44
Lysine	n.d.	n.d.	0.07 ± 0.01	0.05
Histidine	n.d.	n.d.	n.d.	n.d.
Arginine	n.d.	n.d.	n.d.	n.d.
Total	41.08 ± 2.24	100	134.25 ± 22.24	100

GABA, gamma-aminobutyric acid; n.d., not detected

**Table 2:** Free amino acid contents in fresh Japanese basket clams harvested at the Memanbetsu, Yobito, and Katayama areas of Lake Abashiri.

Amino acid	Memanbetsu		Yobito		Katayama	
	mean ± standard deviation (mg/10 g edible portion)	% of total amino acids	mean ± standard deviation (mg/10 g edible portion)	% of total amino acids	mean ± standard deviation (mg/10 g edible portion)	% of total amino acids
Taurine	0.17 ± 0.15	0.65	0.49 ± 0.04	1.65	0.41 ± 0.30	1.38
Aspartic acid	1.39 ± 0.25	5.41	1.22 ± 0.12	4.08	1.59 ± 0.31	5.31
Threonine	1.18 ± 0.08	4.56	1.63 ± 0.15	5.48	1.53 ± 0.55	5.10
Serine	0.82 ± 0.05	3.18	0.78 ± 0.16	2.62	0.96 ± 0.09	3.19
Asparagine	n.d.	n.d.	n.d.	n.d.	n.d.	n.d.
Glutamic acid	5.10 ± 0.81	19.80	6.08 ± 0.14	20.42	5.63 ± 1.30	18.80
Glutamine	1.31 ± 0.26	5.09	0.17 ± 0.08	0.57	0.60 ± 0.48	2.02
Proline	n.d.	n.d.	n.d.	n.d.	2.17 ± 3.75	1.61
Glycine	1.17 ± 0.11	4.56	2.14 ± 0.38	7.18	1.84 ± 0.61	6.15
Alanine	5.44 ± 0.32	21.11	7.07 ± 1.07	23.76	6.50 ± 1.68	21.68
Citrulline	n.d.	n.d.	n.d.	n.d.	n.d.	n.d.
Valine	2.46 ± 0.61	9.55	3.06 ± 0.22	10.29	2.47 ± 0.87	8.23
Cystine	0.61 ± 0.39	2.36	0.41 ± 0.43	1.37	0.36 ± 0.12	1.19
Methionine	0.24 ± 0.42	0.95	n.d.	n.d.	n.d.	n.d.
Isoleucine	1.13 ± 0.42	4.40	1.49 ± 0.13	4.99	1.50 ± 0.65	5.00
Leucine	0.80 ± 0.21	3.11	0.34 ± 0.18	1.13	0.42 ± 0.21	1.39
Tyrosine	n.d.	n.d.	n.d.	n.d.	n.d.	n.d.
β-alanine	2.12 ± 0.43	8.24	3.39 ± 0.42	11.40	4.05 ± 0.83	13.52
Phenylalanine	n.d.	n.d.	n.d.	n.d.	n.d.	n.d.
GABA	1.13 ± 0.21	4.38	1.37 ± 0.11	4.59	1.63 ± 0.24	5.44
Ornithine	0.68 ± 0.04	2.65	0.13 ± 0.02	0.43	0.45 ± 0.29	1.51
Lysine	n.d.	n.d.	n.d.	n.d.	0.03 ± 0.05	0.10
Histidine	n.d.	n.d.	n.d.	n.d.	n.d.	n.d.
Arginine	n.d.	n.d.	n.d.	n.d.	n.d.	n.d.
Total	25.76 ± 2.19	100	29.77 ± 1.35	100	29.97 ± 7.12	100

GABA, gamma-aminobutyric acid; n.d., not detected

## 2 Experimental design, materials and methods

### 2.1 Design

Japanese basket clams (*Corbicula japonica*) are harvested commercially in Japan. Lake Abashiri, in Hokkaido, is connected to the sea of Okhotsk by Abashirigawa River, which is near the northern limit of the species [1]. The free amino acid contents and composition in the edible portion of clams are important taste determinants [2]. In this study, we analyzed the free amino acid contents of clams harvested at three different locations in Lake Abashiri in November. Since the clams of Abashirigawa River are harvested only for a limited period in summer at the Sancho area of the river, they were sampled from the end of June to the beginning of July. Lake clams were also sampled during this period for comparison.

### 2.2 Materials

Japanese basket clams were harvested at the Yobito area of Lake Abashiri and the Sancho area of Abashirigawa River from June to July 2015, and stored in a freezer. The clams were also harvested from three areas (Memanbetsu, Yobito, and Katayama) of Lake Abashiri in November 2015. To flush out the sand, the clams were immersed in 3.5% salt water overnight. The shell was opened by cutting with a jigsaw. Ten grams of the edible portion was homogenized with distilled water using a stomacher, and then the sample tube was filled up to 20 ml with distilled water.

### 2.3 Amino acid analysis

The sample was mixed with same volume of 5% trichloroacetic acid (TCA) to precipitate the proteins contained in the sample solution. After centrifugation (10,000 rpm, 10 min) the supernatant was mixed with same volume if n-hexane and mixture was allowed to stand for a few minutes after which it was filtered through a cellulose acetate membrane (0.2 µm). The amino acid concentration in the filtrate was determined using an amino acid analysis system, LaChrom Elite (Hitachi High-Technologies Corp., Japan) with a post-column ninhydrin method as previously reported [3]. The temperature for ninhydrin reaction was set to 130 °C (reagent flow rate: 0.25 ml/min). The reacted amino acids were detected by recording the absorbance at 570 nm.
